# Mr. Pulley's Account of a Case of Monstrosity

**Published:** 1799-05

**Authors:** John Pulley

**Affiliations:** St. Thomas's Hospital


*To the Editors of the Medical and Physical Journal.*

GENTLEMEN,

PERHAPS the following case of monstrosity may not be unworthy a page in your Physical Journal, nor unacceptable to its numerous readers.

About a fortnight ago, I was called to a woman who was delivered of a female child, full-grown, and, to all appearance, well-formed in eery part, excepting the head, and there was a deformity very remarkable:—Both parietal bones were entirely wanting, and the whole of the frontal bone, except its orbitar processes, and that part which forms the nasal process: the upper part of the squamous portion of the temporal bone (on both sides) was wanting, but the mastoid and zygomatic processes could be distinctly traced, and the meatus auditorius externus seemed to be perfect; as much of the occipital bone as is extended to rather beyond the crucial ridge was present, and the whole length of the upper edge was turned remarkably outward; the integuments on the os occipitis were, from the upper part of the neck, folded up in great quantity, and terminated by dipping down under the edge of the occipital bone, where the hairs were very numerous. As to the brain, there appeared but little, not more in quantity than the half of a common sized orange, which was unequally divided by a longitudinal fissure: I would rather substitute the word excressence for brain, for certainly the appearance had not much the character of the latter—no dura mater existed, and I believe, no pia mater (at least, none on the surface; nor, in fact, could this be expected, there being no longitudinal sinus, into which the vessels could empty themselves, nor was there any appearance of vessels). Pressure on this excrescence seemed to produce no effect; but on this I would not rest, as it certainly could not, in justice, be carried to an extent sufficient to give a decided opinion. The covering seemed only a common cuticle, which was, the day after the birth, abraded in several parts. The child lived about thirty hours, making continually a moaning noise, and breathing with extreme difficulty; at intervals it was seized with a general spasm, in which which state it remained, to appearance dead, for above a minute, when it revived, by sudden and repeated gasps; in such a spasm it was carried off. The child took a little nourishment more than once, swallowing with much difficulty, which was heightened by the want of the palate. Neither urine nor fæces were evacuated. I much lament that I was not able to preserve this curious phenomenon: I endeavoured by various means to persuade its friends to resign it, but their low prejudices resisted every application.

I am, Gentlemen, your's respectfully,

*St. Thomas's Hospital, April* 16, 1799. JOHN PULLEY.

